# Does exposure within an experiment affect the influence of familiar parts versus wholes on figure assignment?

**DOI:** 10.3758/s13414-025-03179-3

**Published:** 2026-01-07

**Authors:** Colin S. Flowers, Mary A. Peterson

**Affiliations:** 1 Department of Psychology, University of Minnesota, Minneapolis, MN USA; 2 Department of Psychology, University of Arizona, Tucson, AZ USA; 3 Cognitive Science Program, University of Arizona, Tucson, AZ USA

**Keywords:** Familiarity, Figure-ground perception, Perceptual organization, Object perception, Past experience, Object detection, Repetition, Familiarity, Parts and wholes

## Abstract

Recurring questions in visual perception concern the role of experience and the relationship between parts and wholes. We investigated these questions in an experiment in which observers reported where they perceived a figure relative to a central border in bipartite displays in which the region on one side depicted either: (1) an identifiable configuration of a familiar basic-level object in its typical upright orientation (a “whole” familiar configuration); (2) an inverted version of (1); (3) a part rearranged (PR) version of (1) comprising the same parts as the upright familiar configuration; (4) an inverted version of (3). Previous research showed that regions are perceived as figures more often when they depict upright familiar configurations rather than inverted or PR configurations, supporting claims that familiar configurations are figural priors, but familiar parts are not. Here, displays depicting the four configuration types were counterbalanced over four trial blocks to elucidate the role of parts versus wholes in figure assignment and to examine whether previous experience with one configuration altered performance with another. Results showed that inverted familiar configurations were weaker figural priors than upright, but were stronger than PR configurations, revealing the importance of inter-part connectivity. Moreover, a reliable effect of block was observed for all configurations, revealing a role for familiar parts in figure assignment. Finally, no influence of the type of configuration viewed in block 1 was observed for any configuration in block 2. We attribute the pattern of results to neural populations representing parts and wholes of well-known objects.

**Supplementary Information:**

The online version contains supplementary material available at 10.3758/s13414-025-03179-3.

## Introduction

An important topic of investigation within the study of visual perception in general and object detection in particular has been when and how past experience exerts an influence (e.g., Peterson, 2019; Peterson & Gibson, 1994a; Vecera & O’Reilly, 1998; Wallach, 1949; Wallach et al., 1953; Wertheimer, 1923; Zuckerman & Rock, 1957). The term “past experience” broadly encompasses many types of experience ranging from repeated presentations within an experiment to effects of learning over longer time periods such as those during which basic-level object categories are formed. Investigations of both types of experience within the same experiment are rare. Moreover, there are abiding questions concerning how representations of wholes and parts interact (Bartolomeo et al., 2015; Gerhardstein & Olsen, 2020), yet attempts to understand how experience affects representations of configurations (“wholes”) versus the “parts” subsumed within a configuration are lacking. In the current study, we address these questions by examining figure-ground perception.

*Figure-ground perception* is one possible perceptual *outcome* when two abutting regions in the visual input share a border. It entails the selection of one side as a shaped entity – a *figure* (i.e., an *object*)*.* The region on the other side of the border is perceived to continue behind the figure as a locally shapeless back*ground*. Other outcomes are possible (e.g., the border could be perceived as a joint between two surfaces or the boundary between two areas on a two-dimensional surface). Figure-ground perception entails object detection; the other outcomes do not (cf., Peterson & Campbell, 2023; Skocypec & Peterson, 2022; Xue et al., 2025).

**Fig. 1:**
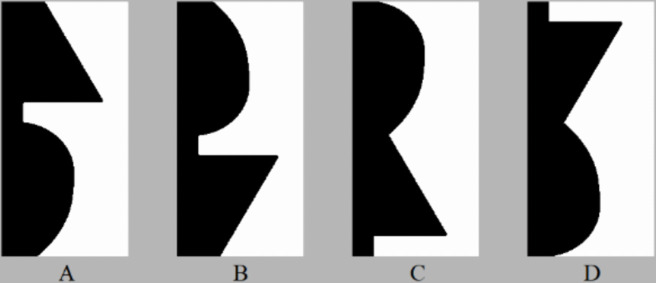
Sample bipartite stimuli. Here, the *critical region* depicts a portion of a table lamp in black on the left in (**A**) the typical upright orientation, (**B**) the inverted orientation, (**C**) the upright novel part-rearranged version, and (**D**) the inverted novel part-rearranged version. Viewers are more likely to perceive the critical region as figure in (A) than (B‒D)

There is substantial evidence that past experience, operationalized as configurations of well-known objects, influences figure assignment. For instance, Peterson and Gibson (1994a) asked participants to report which of two abutting regions they first perceived as figure within a briefly presented bipartite display followed by a mask. The region on one side of a central border was a *critical region* in that at the border it suggested a portion of a well-known, *familiar*, basic-level object with a canonical upright orientation. Other factors were equated across the two regions. Their participants were more likely to perceive the critical region as figure when it suggested the familiar object in its upright orientation rather than an inverted orientation (see Fig. 1A and B). The orientation change held constant other factors known to influence figure assignment, yet for objects with a typical upright orientation, the upright configuration was more familiar than the inverted one. Hence, such results were interpreted as evidence that *familiar configuration* is a factor that predicts where figures lie relative to a border; that is, a figural “prior”. Familiar configuration effects on figure-ground perception have been replicated many times in both long and short exposures (e.g., Barense et al., 2012; Gibson & Peterson, 1994; Navon, 2011; Peterson et al., 2000; Peterson et al., 1998; Peterson & Gibson, 1994b; Ralph et al., 2014; Skocypec & Peterson, 2022; Vecera & Farah, 1997;Vecera & O’Reilly, 1998; see Peterson, 2019, for review).

To further investigate whether these familiarity effects were due to familiar *configuration*s or to familiar parts, novel part-rearranged (PR) critical regions were created by dividing the upright familiar configurations into parts at the minima of curvature and spatially rearranging the parts into a novel configuration (upright novel PR configurations; see Fig. 1C). Peterson et al., (Barense et al., 2012; Gibson & Peterson, 1994; Peterson et al., 1998; 1991) failed to find effects of familiarity when neurotypical observers viewed bipartite displays in which the critical regions depicted upright novel PR configurations. Thus, at least when embedded in a novel configuration, familiar *parts* did not seem to affect figure assignment; hence, these experiments were taken as further evidence that familiar *configurations,* not *familiar parts,* are a prior for figure assignment.

Barense et al. (2012) found that damage to the perirhinal cortex (Prh cortex) can unveil effects of familiar parts on figure assignment independent of configuration, however. Individuals with Prh cortex damage failed to perceive critical regions portraying familiar configurations as figure more often than regions comprising the same parts rearranged into novel configurations*:* Compared to controls, they reported perceiving critical regions depicting upright (but not inverted) novel PR configurations as figure more often and critical regions depicting upright familiar configurations as figure less often. These results demonstrated that an intact Prh cortex plays a role in the typical advantage for familiar configurations over familiar parts in figure assignment.^1^ Cacciamani et al., (2017; Peterson et al., 2012) presented functional magnetic resonance imaging (fMRI) evidence consistent with the view that, for neurotypical observers, the Prh cortex plays a role in reducing effects of familiar parts in upright novel PR configurations on figure assignment: Measuring BOLD (blood oxygen level-dependent) responses, they observed that, compared to Prh cortex responses to control inverted novel PR configurations (see Fig. 1D), Prh cortex responses were higher for upright familiar configurations and lower for upright novel PR configurations. These results suggest that the Prh cortex detects the mismatch between the familiarity of the parts and the configuration in upright novel PR configurations; otherwise, the BOLD response in the Prh would not have been lower for the upright than the inverted novel PR configurations. These fMRI results also suggested that familiar parts activate familiar configurations even when they are embedded in novel configurations: V2 responses to familiar parts mirrored the Prh responses, consistent with the view that feedback from the Prh modulated lower-level responses and suggesting a mechanism for the absence of any effects on figure assignment of familiar parts in upright novel configurations.

The results of a priming experiment Cacciamani et al. (2014) conducted with neurotypical observers also suggest that familiar configurations are implicitly activated by their parts even though embedded in novel configurations: They found that upright novel PR configurations shown before bipartite displays primed representations of the upright familiar configurations from which they were created. The results did not depend on whether the upright PR novel configurations were sketched on the figure or the ground side of the border of the prime. Given that the ground side of a border appears shapeless, the organization-independence of these results implies that upright familiar configurations are activated by upright parts in a novel configuration regardless of whether the novel configuration was consciously perceived.

The research reviewed above raises the question of whether previous experience with a stimulus depicting a novel PR configuration within an experiment affects responses to a stimulus depicting a familiar configuration viewed later and vice versa: Does viewing a stimulus depicting a familiar configuration alter the perceived organization of stimulus depicting a novel PR stimulus comprising the same parts viewed later, or vice versa? In previous studies, displays with critical regions depicting upright familiar configurations were typically shown once only intermixed with *either* displays in which critical regions depicted novel PR configurations or inverted familiar configurations that were also shown once only. The order of presentation of the familiar configuration and its variation was counterbalanced to attempt to eliminate effects of the prior presentation of one configuration type on the effectiveness of another configuration type. Order effects were not observed, but small numbers of displays and participants were tested. Moreover, inverted familiar configurations and novel PR configurations were not tested in the same experiment, so the role of properly interconnected parts (as in an inverted whole) versus improperly connected upright parts was not examined. Therefore, questions remain regarding whether the prior presentation of various variations of familiar configurations within an experiment influences figure reports for other configurations comprising the same parts. For instance, does the prior presentation of an upright novel configuration comprising familiar parts on a previous trial increase the influence of the familiar configuration comprising the same parts on a subsequent trial? Or does the prior presentation of a familiar configuration prime the familiar parts such that they serve as figural priors even when embedded in a novel or inverted configurations? The answers to these questions can reveal the mechanisms of familiar part and whole effects on figure assignment. Furthermore, the answers are practically important for experiments using these displays.

To address these questions, in the present experiment, we counterbalanced configuration type over four blocks of trials and tested a large number of participants (see *Methods*). In addition to exploring whether prior experience with critical regions depicting one type of configuration affects figure assignment in displays with critical regions depicting another type of configuration, the present experiment will reveal whether repeating the collection of parts over four blocks of trials, albeit in different configurations, affects figure assignment; this is because all variations comprise the same parts.

Because repetition can alter performance in other types of experiments particularly by increasing reliance on features rather than wholes (cf. Skocypec & Peterson, 2022), we avoided repeating individual stimuli in previous experiments using bipartite displays. We avoid repetition of a particular configuration in the current experiment as well (see *Methods*). The results will provide information about the role of parts and wholes in figure assignment, a question that has not been addressed systematically. The observed answers will be important for understanding the mechanisms of part and whole perception, a recurring theme in visual perception research.

In addition to their theoretical importance, the questions addressed in the current experiment are practically important because a limited number of bipartite displays exist. If we find that the pattern of results is the same regardless of presentation order, that would suggest that these stimuli can be used in designs that require more trials. Skocypec and Peterson (2022) recently argued that figure assignment is an ideal index of object detection since, without figure assignment, patterns exist, but objects to not. Hence, it would be good if these stimuli can be used in designs requiring many trials. Here, to elucidate the roles of familiar parts and familiar wholes in object detection, as indexed by figure assignment, we present all four variants of a familiar configuration to individuals in counterbalanced order and examine both average effects and order effects.

## Current study

To investigate how contributions to figure assignment from familiar “parts” and “wholes” (where the latter are learned object categories) are affected by the prior presentation of different configurations comprising the same parts, we probed participants’ reports of which of two abutting regions they perceived as figure across four blocks of trials using bipartite stimuli where the critical region depicted upright intact, inverted intact, novel upright PR, or novel inverted PR configurations. Flowers et al. (2020) recently published identification norms for the critical regions of upright familiar configurations and upright and inverted novel PR configurations. Although the various configurations differ in the extent to which participants agree they depict familiar objects (Flowers et al., 2020), they are constructed with the same segmentations of the central contour of a “source” upright familiar configuration. Thus, the “parts” delimited between successive minima of curvature do not change across the configurations, but object familiarity – derived from the configural “whole” – varies greatly. Here, the different configurations of each stimulus are presented to participants across blocks in different orders. The parts are repeated across blocks (sometimes upright and sometimes inverted) given that they are present in all configuration types. The stimuli denote semantically familiar configural “wholes” most strongly only in their upright configuration which is presented only once. Thus, these stimuli allow us to investigate how contributions to object detection from familiar “parts” and “wholes” are affected by repetition of parts but not wholes across four blocks of trials.

### Methods

#### Participants

Participants were 122 University of Arizona undergraduate students with reported normal or corrected-to-normal vision, who participated for course credit or monetary compensation. The experiment was designed for 96 participants: four participants for each of 24 unique versions to accommodate balanced stimulus and presentation order (see *Stimulus and presentation balancing*). A formal power analysis was not conducted; the number of participants was selected based on previous studies. For instance, Peterson and Gibson (1994a, 1994b) examined figure judgments with 24 participants in each of their experimental conditions and other studies indexing figure ground judgments used similar numbers (Lass et al., 2017; Peterson & Enns, 2005).

The data from four participants were lost due to system malfunction. One participant was excluded for failing to understand the instructions, and one participant for reporting that they could not see the bipartite display. The data from five participants were excluded from analyses for failure to perform the task properly; these participants reported searching for either the black or the white side rather than making a figure judgment (see post-experiment questionnaire under *Methods*). These exclusion criteria were a priori; the data from these participants were never viewed. Exclusions based on outliers and response thresholds are described in the *Data analysis* section. The experiment was run for 96 participants at which point the above exclusions were applied. Where exclusions were necessary, replacement participants were tested in the same program as the excluded participant until the final sample (96 participants; four in each of 24 unique programs) was collected. Final analyses are based upon responses from 96 participants.

#### Apparatus and stimuli

##### Apparatus

The experiment was run on a computer with an Intel®Core i7-4790 CPU running at 3.60 GHz and an AOC G2460PG 24 class Nvidia G-Sync LCD gaming monitor (29.68° × 17.06°) running at 100 Hz. The experiment was run using MATLAB (2015a; The MathWorks Inc., Natick, MA, USA) and the Psychophysics toolbox extension (Brainard & Vision, 1997; Kleiner et al., 2007). Responses were collected using an Adesso EasyTouch 220 mechanical numeric keypad connected through the ps/2 port. The buttons on the keypad were physically removed except for two buttons, one on the left side of the keypad and one on the right. These two buttons were used by participants to make their figure judgment (whether they perceived the left or right region as the figure). A foot pedal was used to initiate trials and navigate through instruction slides.

##### Stimulus and presentation balancing

The stimuli were 152 bipartite stimuli: the region on one side of this border was white (rgb = [255 255 255]; 87.33 foot lamberts [fL]); the region on the other side was black (rgb = [0 0 0]; 0.13 fL). One of the two regions was a critical region in that it depicted one of the four types of configurations: (1) Upright intact familiar configurations (see Fig. 1A). At least 75% of pilot participants agreed on the identity of this object; mean agreement was 89.6% (Flowers et al., 2020; see Appendix A for a list of the objects depicted by the intact upright familiar configurations). These 38 stimuli were the “source” stimuli from which the other three configuration types were created. (2) Upside-down (Inverted) versions of the intact familiar configurations (see Fig. 1B). (3) Upright novel Part-rearranged configurations (upright novel PR; see Fig. 1C). Agreement among pilot participants on the identity of objects depicted by upright novel PR displays was much lower (mean: 41.2%; Flowers et al., 2020); Moreover, the identity they perceived was rarely the same as that of the familiar configuration (see Flowers et al., 2020). (4) Inverted versions of the upright novel PR stimuli (see Fig. 1D). In these inverted novel PR displays the configuration is novel and the parts are less familiar than in the upright PR novel displays because part familiarity is learned coincidently with configuration familiarity. Mean agreement among pilot observers was 32.9% for inverted novel PR displays and the identity they perceived was rarely the same as that of the familiar configuration (see Appendix A). We did not ask pilot participants to identify the object depicted in inverted intact displays because we sought to determine the identifiability of the depicted objects in retinal coordinates and were concerned that once participants discovered some inverted familiar objects, they would thereafter attempt to mentally rotate the stimuli to upright before responding.

To examine whether experience with one configuration type affected figure reports with another configuration type, 38 source stimuli depicting familiar configurations were split into four subsets (A‒D – two subsets of nine stimuli and two subsets of ten stimuli). For each participant the four subsets of stimuli were presented in each of four blocks of 38 trials such that each configuration derived from one source stimulus was shown once only across the blocks (see columns in Table 1) and different configurations derived from one source stimulus appeared in different blocks (see rows). Block order was counterbalanced such that each configuration type was shown in a block preceding and following every other configuration type equally often.

**Table 1:** Latin square used to assign the four subsets of stimuli (A‒D) to different configuration conditions across blocks. Columns indicate the configuration in which the subsets were viewed in each block in one program

		Configuration			
		Intact	Inverted	Upright Part-Rearranged	Inverted Part-Rearranged
Block	1	A	B	C	D
	2	B	D	A	C
	3	C	A	D	B
	4	D	C	B	A

Table 1 illustrates a program in which stimuli in subset A were shown in the upright intact configuration in block 1 and in the upright novel PR configuration in block 2; stimuli in subset B were shown in the inverted configuration in block 1 and in the intact configuration in block 2; stimuli in subset C appeared in the upright PR configuration in block 1 and in the inverted PR configuration in block 2; and stimuli in subset D appeared in the inverted PR condition in block 1 and in the inverted condition in block 2.

For block two, the four subsets of stimuli were presented such that each configuration type presented in block one was followed by each of the three other configuration types equally often (e.g., of the 96 participants who viewed a bipartite display depicting a given Upright Intact familiar configuration in block one, 32 viewed displays depicting each of Inverted intact, upright novel PR configurations, and inverted novel PR configurations in block two). Thus, tests of whether the type of configuration depicted in the bipartite display viewed in block one affected figure reports for the different configuration type viewed in block 2 were based on 32 participants. Balancing which configuration type followed the others continued in blocks three and four, resulting in 24 different programs (see Online Supplementary Materials). The divisions into subgroups of participants in those later blocks became too small to allow us to compare performance as a function of the different configurations viewed previously, however. Moreover, it was not possible to predict what influence viewing two different configurations would have on performance of another configuration. Hence, we analyzed the effect of previously seeing a different configuration on performance with a given configuration type in a between-participants analysis of whether performance in block two varied with configuration type viewed in block 1.

The 38 stimuli were presented in a random order in block one. Stimuli from a given source object that were presented in the first 19 trials of block one were presented in different configurations in random order in the first 19 trials of each subsequent block. Likewise, stimuli that were presented in the second half of block one (trials 20‒38) were presented in a different configuration in random order in the second half of trials in each subsequent block. Thus, there were always at least 19 trials (range: 19‒55; mean of 37 trials) before a different configuration of a given stimulus was repeated in the next block. Hence, we do not examine effects of very recent experience in this experiment. Within-participants analyses of performance across blocks by stimulus type are based on data from 96 participants (24 participants per each of the four stimulus subsets). Mean performance across blocks was assessed to determine whether the means for the four types of configurations were affected by having seen other variants in earlier blocks.

The four subsets of stimuli were also balanced within and across participants for the side and contrast of the critical region. Within each of the subsets of ten stimuli, the critical regions were presented on the left of the central border in half (five) and on the right in half (five). One subset of nine stimuli contained a subgroup of five stimuli in which the critical region was on the left of the central border and a subgroup of four stimuli in which the critical region was on the right. The other subset of nine stimuli contained a subgroup of four stimuli in which the critical region was on the left of the central border and a subgroup of five stimuli in which the critical region was on the right. Hence, across all stimuli, the left/right location of the critical region relative to the central border was equated. Whether the critical region was presented in black or white was balanced in the same manner but with different subgroupings of stimuli within each set. The result of this balancing was that, for a given participant, the critical regions were equally likely to be presented in every combination of contrast and side. For a given participant, all configurations derived from the same source configuration were presented in the same contrast and on the same side. Across participants, the stimuli were placed in different subgroups, such that the critical region for each stimulus was equally likely to be presented in every combination of contrast and side.

##### Masks

Masks were custom made for bipartite displays; a unique mask was used for each trial; 152 masks were used during experimental trials and 16 were used during practice trials. Of the masks used during experimental trials, four were randomly assigned to each of the four configurations of each stimulus. Masks were created from images of multiple bipartite stimuli (see Appendix B for information on mask creation). Template images were created using the central borders of the bipartite stimuli. These borders were placed along a horizontal image such that they separated alternating black and white regions. These horizontally elongated images were then segmented into 50 × 50 pixel boxes and the boxes were shuffled to create masks. This shuffling procedure was repeated such that a unique mask was used on each trial (cf., Skocypec & Peterson, 2022).

#### Procedure

Participants were tested individually. They first signed a consent form that was approved by the Institutional Review Board (IRB) of the Human Subjects Protection Program at the University of Arizona; the IRB also approved our protocol. Instructions were presented on the computer screen; participants followed along as the experimenter read them aloud. They were introduced to figure assignment and shown examples of enclosed figures first, and then of a bipartite stimulus (not used in the experiment). Participants were instructed to report which of the two regions first appeared as the figure. It was stressed that this was a judgment, and that their judgments would not be considered “correct” or “incorrect.” Participants then completed 16 practice trials. Practice trials contained stimuli of different configurations (upright, inverted, PR, and inverted PR) that were not used in the experiment. There was a break after the first eight practice trials, during which participants could ask any clarifying questions. Following the practice trials, the participants were left alone to complete the experimental trials. There were four blocks of 38 trials with a break after each block. Participants were also instructed that they could take a break at any time they chose, because a given trial would not start until they pressed the foot pedal.

**Fig. 2:**
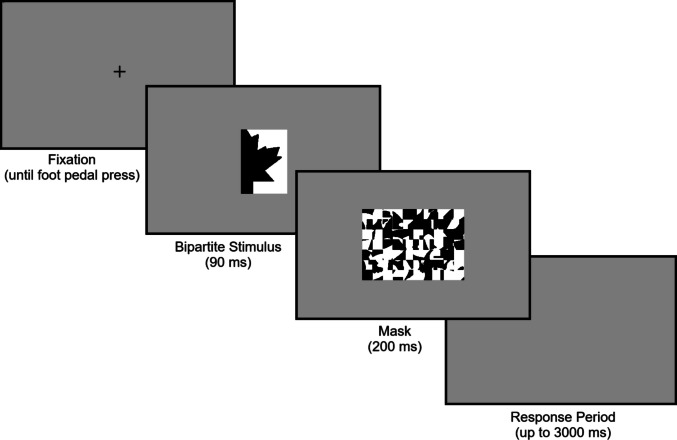
Trial structure. The bipartite stimulus shows the upright configuration of a maple leaf on the left of the central border in black. The complementary region, matched in area, is white and on the right

All stimuli were presented on a medium gray background (rgb = [182 182 182]; 45.70 fL). Each trial (practice and experimental) began with a black fixation cross (0.79° × 0.79°; rgb = [0 0 0]; 0.13 fL) in the center of the screen. Participants were instructed to look at the fixation cross, and when they were ready, to press the foot pedal to initiate the trial. All stimuli were presented at fixation. On each trial, a bipartite stimulus (5.43° High × 1.76° – 5.54° Wide) was presented for 90 ms and followed by a mask (7.91° H × 5.54° W) for 200 ms. Participants were instructed to indicate which region (left or right) they first perceived as the figure in the bipartite stimulus by pressing a right or left button on a button box. Participants had up to 3,000 ms from the offset of the mask to make their response (see Fig. 2). If they failed to respond within this time period, the trial was marked as a “timeout,” and no response was recorded.

##### Post-experiment questionnaire

After the experimental trials were completed, participants were questioned about their task performance to confirm that they were making figure judgments regarding the bipartite stimuli. They were asked what strategy they were using to make their responses. They were also asked to estimate the percentage of trials on which they chose the black side, and the same for the white side. If a participant indicated that they chose either the black or white region as figure a very high percentage of the time (> 80%), they were asked follow-up questions about their strategy. If they indicated that mostly they chose black or white because they perceived it as figure, they were not excluded. However, if a participant indicated that they choose either black or white more often because they were searching for black or white figures, their data were excluded from analysis, because they were making biased judgments about the stimuli.

#### Data analysis

Data from 111 participants were entered into data analyses. The data from two participants were excluded because they failed to respond on at least 85% of trials. Failing to respond to a trial was defined as either a timeout (see *Methods*) or a response within 150 ms of the onset of the bipartite stimulus. The data from an additional 13 participants were removed from subsequent analyses because they were outliers in that the mean percentage of trials on which they reported perceiving the critical region as figure in the upright familiar configuration displays was more than two standard deviations from the sample mean.^2^ Only performance in the upright configuration condition was used to identify outliers as repeated outlier removal on all configurations would inflate the percentage of excluded participants, thus reducing generalizability. The upright configuration was chosen because the effects of familiarity on figural judgments are strong and well documented. The final sample consisted of 96 participants. All analyses were run using JASP (0.18.3.0; JASP Team, 2024).

### Results

**Fig. 3:**
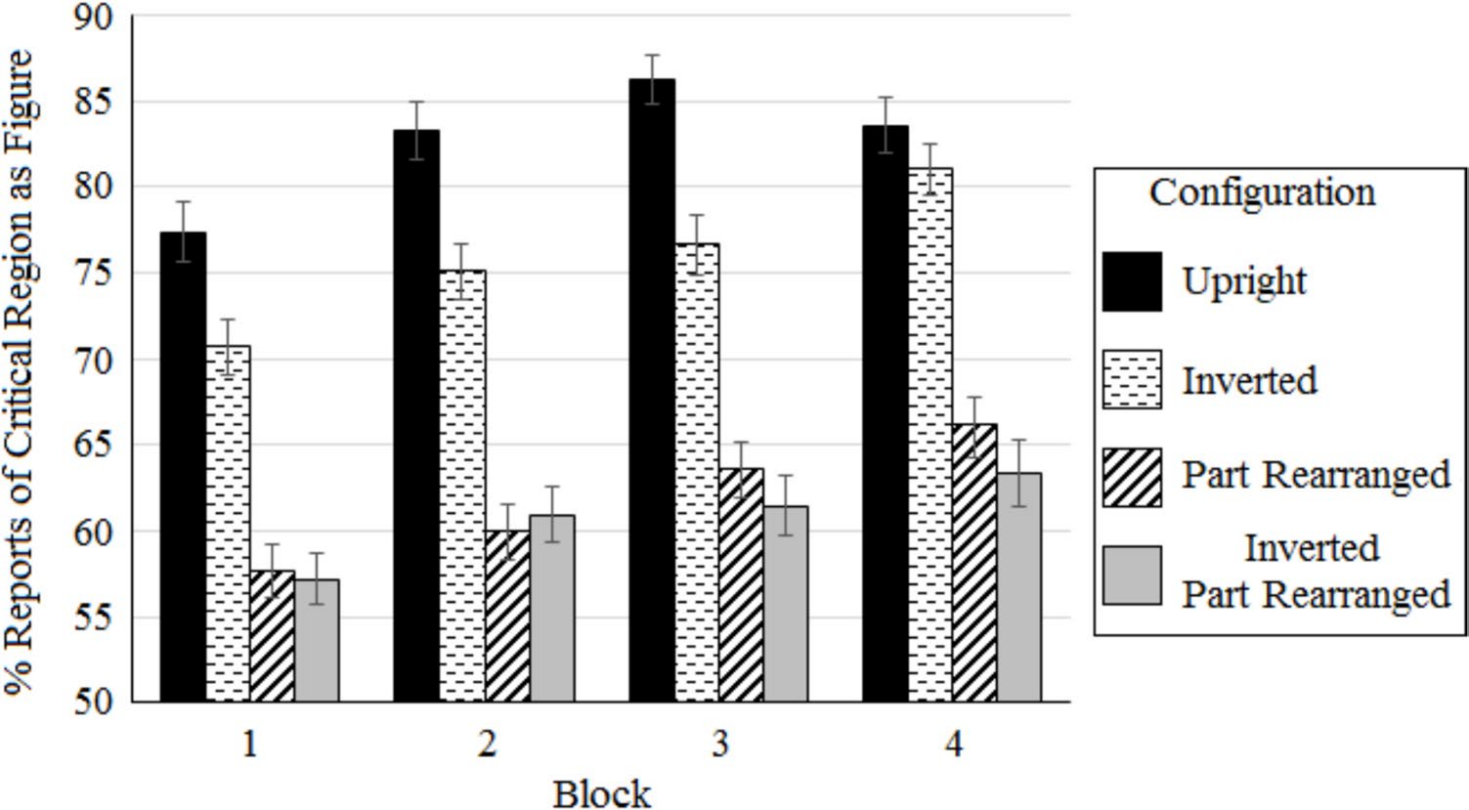
Mean percentage of critical regions perceived as figure by block and configuration. Error bars represent SEM

To examine the effect of exposure to the stimuli in different configurations across blocks, a 4 (configuration depicted by the critical region: upright intact, inverted intact, upright novel PR, inverted novel PR) × 4 (block: 1‒4) repeated-measures ANOVA was run on individuals’ mean percentage of critical region as figure reports (see in Fig. 3). Mauchly’s test of sphericity was significant for block, *Χ*^2^(5) = 12.799, *p* = 0.025, but not for configuration, *Χ*^2^(5) = 9.759, *p* = 0.082, or the interaction, *Χ*^2^(44) = 60.356, *p* = 0.051. Huynh–Feldt estimates of degrees of freedom were used to correct for heteroscedasticity for block (ε = 0.943). The results are shown in Fig. 3.

A significant main effect of configuration was obtained, *F*(3,285) = 254.884, *p* < 0.001, ${\eta }^{2}= 0.288$. There were significant differences in mean percentage of critical region as figure reports between each pair of configurations except for upright and inverted novel PR configurations. The highest mean percentage of critical region as figure reports (82.6%) was obtained for bipartite displays depicting upright intact configurations; this was significantly greater than the next highest – bipartite displays depicting inverted intact configurations (75.9%), *t*(95) = 7.115, *p*_*holm*_ < 0.001, d = 0.682. Critical regions were perceived as figures more often in bipartite displays depicting inverted intact configurations than in those depicting both upright novel PR configurations (61.9%), *t*(95) = 14.717, *p*_*holm*_ < 0.001, d = 1.53, and inverted novel PR configurations (60.7%), *t*(95) = 15.919, *p*_*holm*_ < 0.001, d = 1.510. There was not a significant difference between critical region figure reports for upright and inverted novel PR configurations, *t*(95) = 1.203, *p*_*holm*_ = 0.230.

A significant main effect of block was also obtained, *F*(2.828, 268.683) = 17.390, *p* < 0.001, ${\eta }^{2}= 0.029$. This effect was best explained by a linear contrast, t(95) = 5.938, *p* < 0.001, showing that reports of the critical regions as figure increased over blocks. This increase tapered off over blocks: Mean percent critical region as figure reports averaged over configuration type were 65.7%, 69.8%, 72.0%, and 73.6% across blocks 1‒4, respectively. This tapering off may be because figure assignment reached a functional ceiling.

The interaction between block and configuration was not significant, *F*(9,855) = 1.054, *p* = 0.395, showing that presenting variations of a source stimulus across four blocks did not change the pattern of responses across configurations. The lack of a significant interaction between block and configuration was probed further by a Bayesian repeated-measures ANOVA (JASP 0.18.3.0). The results were the same, with the favored model including main effects of block and configuration, BF_10_ = 1266.665. The model including the interaction of block and configuration produced a Bayes factor of BF_inclusion_ = 0.013, providing extreme evidence against including the interaction. The absence of an interaction between block and configuration supports the conclusion that part familiarity underlies the main effect of block because only familiar parts are repeated across blocks. Thus, this study revealed a previously undocumented influence of familiar parts on figure assignment in neurologically intact participants, an influence that increases across blocks for all configurations.

To further investigate potential effects of exposure to one configuration on perception of another configuration, we examined block-two figure reports with stimuli depicting each configuration type as a function of which configuration derived from the same source stimulus participants had viewed in block one. Recall that participants had viewed bipartite stimuli comprising the same parts depicting only one of the other three configuration types in block one. Four between-participant ANOVAs were run on the mean percentage of critical regions perceived as figures in block two, one for each configuration type (shown in the rows of Table 2). The factor for each ANOVA was which of the three other configurations created from the same source stimulus was presented in block one (shown in the columns in Table 2).

**Table 2:** Percentage of critical regions perceived as figure in block two as a function of the configuration of the same source stimulus viewed in block one

		Block-one configuration			
		Upright Intact	Inverted Intact	Upright Novel PR	Inverted Novel PR
Block-two configuration	Up. Intact		82.07 (3.30)	82.93 (2.77)	84.90 (2.63)
	Inv. Intact	76.04 (2.55)		77.01 (2.35)	72.19 (3.54)
	Up. Novel PR	61.10 (2.80)	60.34 (2.88)		58.47 (2.60)
	Inv. Novel PR	64.44 (2.79)	57.01 (2.76)	61.40 (2.82)	

*Up.*  upright, *Inv.*  inverted, *PR* part rearranged.

Standard error of the mean is shown in parentheses.

None of these ANOVAs produced significant differences. Figure reports for a given configuration type in block two were unaffected by the type of configuration participants had viewed in block one. Figure reports regarding bipartite displays with critical regions depicting Upright Intact Familiar Configurations in block two did not differ significantly as a function of whether participants had viewed bipartite displays with critical regions depicting Inverted Intact Familiar, Upright Novel PR, or Inverted Novel PR configurations in block one (range = 82.07‒84.9) *F*(2,93) = 0.247, *p* = 0.782 (first row of Table 2). Likewise, figure reports regarding bipartite displays with critical regions depicting Inverted Intact Familiar Configurations in block two did not differ significantly as a function of whether participants had viewed bipartite displays with critical regions depicting Upright Intact Familiar, Upright Novel PR, or Inverted Novel PR configurations in block one (range = 72.19–77.01): *F*(2,93) = 0.797, *p* = 0.454 (second row of Table 2). Similarly, figure reports regarding bipartite displays with critical regions depicting Upright Novel PR in block two did not differ significantly as a function of whether participants had viewed bipartite displays with critical regions depicting Upright Intact Familiar, Inverted Intact Familiar, or Inverted Novel PR configurations in block one (range = 58.47–61.10): *F*(2,93) = 0.239, *p* = 0.788 (third row of Table 2). And, figure reports regarding bipartite displays with critical regions depicting Inverted Novel PR configurations in block two did not differ significantly as a function of whether participants had viewed bipartite displays with critical regions depicting Upright Intact Familiar, Inverted Intact Familiar, or Upright Novel PR configurations in block one (range = 57.01‒64.44): *F*(2,93) = 1.794, *p* = 0.172 (fourth row of Table 2). Thus, the results failed to support the hypothesis that figure assignment in block two differed as a function of which configuration was viewed in block one. The lack of configuration-specific effects suggests that in future experiments different configurations of these stimuli can be shown without contamination from previous stimulus presentations.

Because these analyses failed to provide significant differences, they were rerun as Bayesian between-participants ANOVAs to see whether the null effects could be interpreted. The Bayesian analyses provided moderate evidence that repetition of the bipartite stimuli does not differentially affect future figure judgments based upon which configuration was previously viewed (Upright Intact BF_10_ = 0.115; Inverted Intact BF_10_ = 0.179; Upright Novel PR BF_10_ = 0.115; Inverted Novel PR BF_10_ = 0.395).

## Discussion

The current study investigated whether the prior presentation of bipartite displays with critical regions depicting different configurations of the same parts affects the degree to which familiar configuration as opposed to familiar parts determines figure assignment. This is a theoretically important question because both behavioral and fMRI evidence suggests that familiar parts may implicitly activate familiar configurations even when they are embedded in novel PR configurations (Cacciamani et al., 2014, 2017; Peterson et al., 2012). Yet previously, for neurologically intact participants, familiar parts did not seem to affect figure assignment, whereas familiar configurations did. Hence, it is important to understand whether this implicit activation affects performance on later trials on which familiar configurations are viewed. Similarly, it is important to know whether the prior presentation of a familiar configuration affects the extent novel PR configurations comprising familiar parts affect figure assignment.

We examined effects of previous exposure within an experiment by presenting four variations of 38 source stimuli to 96 participants across four blocks of trials. We examined first whether the typical pattern of results in which critical regions depicting upright intact familiar configurations are perceived as figure more often than critical regions depicting inverted and part-rearranged variants of the same parts was obtained overall, even though observers had seen and made figure reports regarding different configurations of the same parts in previous blocks. Second, we examined whether a single prior presentation of the parts of a familiar configuration embedded in one configuration affected figure assignment when those parts were next viewed in a different configuration. We examined the same question for all combinations of the same parts in different configurations: For instance, did the prior presentation of a familiar configuration affect figure assignment when the same parts were embedded in an inverted or novel configuration? To address these questions, we counterbalanced presentations of four subsets of the stimuli in the four configurations over blocks.

Results showed that, across blocks, the percentages of critical regions as figures increased *for all configuration types across four blocks of trials: in block 4, mean figure reports were increased 7.9% compared to block one*. There was not a significant interaction between block and configuration, suggesting that repeated presentations of familiar parts in different configurations within an experiment did not affect perceiving the critical regions of any one configuration as figure more than the others.

We infer that increased reports of critical regions as figure arise at the level of the familiarity of parts per se rather than the features of the central border because border features do not favor one side as the figure. One possible explanation of the main effect of block is that participants updated their task set over the course of the experiment to upweight familiarity per se. According to this account it would not matter which configuration of a particular stimulus is viewed in early blocks as the weight of the figural prior of familiarity is increased regardless of the specific stimulus. Other research has shown that participants can alter the weight assigned to familiarity as a figural prior (e.g., Flowers & Peterson, 2018; Ghose & Peterson, 2021). Note that this account assumes that familiar parts are detected in all configurations before figure assignment, which is consistent with previous research in our laboratory and others (e.g., Cacciamani et al., 2014, 2017; Gerhardstein & Olsen, 2020; Peterson et al., 2012). On this view, familiar parts *can* affect figure assignment; the design used here in which individuals viewed four configurations comprising the parts of a familiar configuration across four blocks (i.e., upright intact, inverted, upright, and inverted PR novel) without repetition of any particular configuration showed that familiar parts can serve as priors. It is important to point out, however, that the collection of parts was the same in all configurations although their interconnectivity varied with configuration type. Therefore, the block effects may be attributable to the repetition of *collections of parts*; indeed, the collection may be familiar in inverted and PR displays even though the configuration of parts is not. This possibility requires further investigation, perhaps by recombining the parts of various source stimuli.

As a potential mechanism for the effect of repeating familiar parts or part collections, we suggest a change in threshold of neural populations (NPs) representing the object category depicted by the familiar configuration. Given that all critical regions were derived from an upright source familiar configuration, we assume that all configurations comprising the same parts would activate the neural population (NP) representing the object depicted by the familiar configuration (cf., Perrett et al., 1998). Perrett et al. proposed that objects with a typical upright are represented by NPs that include more memories of upright than inverted versions of that object and still fewer representations of the individual parts of that object. Consequently, evidence accumulates faster in the NP for an intact upright version of an object than for inverted versions or for parts of objects. (See Xue et al., 2025, for recent model explication of how NPs within a semantic network can account for the influence of word labels on object detection.) To our knowledge, there have been no investigations of how the output of such NPs is affected by presentation of intact upright, inverted, and part-rearranged stimuli within an experiment. The present results suggest such as investigation would be worthwhile.

This experiment was the first in which displays with critical regions depicting upright intact and inverted intact familiar configurations were intermixed with displays with critical regions depicting upright and inverted novel PR configurations. We found a higher rate of critical region as figure reports for inverted intact configurations than for upright novel PR configurations. This finding supports claims for the superiority of configuration effects rather than part effects in figure assignment even though effects of repeated presentations of familiar parts were also observed. In addition, the finding that critical regions depicting inverted intact familiar configurations were seen as figure more often than critical regions depicting both upright and inverted novel PR configurations is further evidence that past experience underlies familiar configuration effects: Objects that have a typical upright may have been seen in atypical orientations, but the parts of those objects had not been encountered in a novel configuration.

Next, we investigated more fine-grained questions regarding repetitions of different configurations of the same stimuli. To examine these questions, for each type of configuration we examined figure judgments in block two as a function of which configuration of the same source stimulus was viewed in block one. We found that the first experienced configuration of a stimulus did not influence figure assignment when the same parts were next encountered in a different configuration. This was the case regardless of whether the critical region was perceived as figure in block one. For instance, critical regions depicting upright intact familiar configurations were perceived as figure equally often regardless of whether they were preceded by stimuli depicting an inverted intact configuration, an upright novel PR configuration, or an inverted novel PR configuration. Thus, at least when the block two presentation of a configuration derived from the same source configuration was viewed at least 19 trials after the first, no specific influence of the first presentation remained. In future experiments, it would be interesting to examine whether different carryover effects arise from more recent presentations. Priming has been observed following upright part rearranged displays compared to inverted part rearranged displays (Cacciamani et al., 2014), demonstrating that different configurations can impact later figure judgments at least within a very short time window (less than 500 ms) and within a single trial. Here, no such effects were observed when parts of the same source stimulus were presented in different configurations with many intervening trials. It remains an open question when these effects dissipate, be it a fixed amount of time or number of intervening trials.

In addition to implications for theoretical issues regarding parts and wholes, the results of the present experiment are of practical importance in showing that different variations of a familiar configuration can be repeated across blocks without altering the pattern of results. This is important because these figure reports provide valuable tests of object detection independent of identification (cf. Skocypec & Peterson, 2022) and can reveal effects of both implicit and explicit activation of object categories and their parts.

## Electronic supplementary material

Below is the link to the electronic supplementary material.

Supplementary file1 (PDF 85 kb)

## Data Availability

The bipartite stimuli are available online (https://osf.io/j9kz2/). The remaining data and materials for the experiment are available upon request.

## Appendix A: Familiar Configurations depicted in the Bipartite Stimuli

Anchor

Axe

Butterfly

Bell

Boot

Cow

Dog

Duck

Eagle

Elephant

Face

Faucet

Fire Hydrant

Flower

Foot

Guitar

Hand

House

Lamp

Lightbulb

Maple Leaf

Mickey Mouse

Owl

Palm Tree

Pig

Pineapple

Rhino

Seahorse

Snowman

Teapot

Train

Tree

Toilet

Trumpet

Umbrella

Watering Can

Wine Glass

Woman

## Appendix B: Mask Creation

First, template images were created that consisted of the borders of the silhouettes and contained an equal number of black and white pixels.

These images were segmented into boxes (50 x 50 pixels).

The boxes were then randomly shuffled, and the base image was split into two, creating two masks that contained features of the bipartite silhouettes used.

## Footnotes

1 Barense et al., (2005) and others reported consistent evidence for the role of the Prh in representing configurations in memory.

2 Standard exclusion criteria in our lab are to eliminate participants who respond on less than 85% of trials and to trim outliers based on a cutoff of two standard deviations from the mean; see Flowers and Peterson (2018) and Flowers et al. (2021) where these same criteria are used.

## Code availability

Code for the experiment is available upon request.

## Declarations

### Open practices statement

The bipartite stimuli are available online (https://osf.io/j9kz2/). The remaining data, materials, and code for the experiment are available upon request. The experiment was not preregistered.
