# Supplementary material for: Does exposure within an experiment affect the influence of familiar parts versus wholes on figure assignment?
Source: Atten Percept Psychophys. 2026 Jan 7;88(2):43. doi: 10.3758/s13414-025-03179-3 (PMC12775106; doi:10.3758/s13414-025-03179-3)
Supplement: Supplementary file 1 — Supplementary file1 (PDF 85 kb) [file 13414_2025_3179_MOESM1_ESM.pdf]

**Supplementary Materials**

Table SM 1. Table 1 (in the main manuscript) shows a template, a Latin square, showing how stimulus subgroups were distributed throughout the blocks and configuration conditions of a single version of the experiment. Each letter populating the table (A-D) represents a stimulus subgroup. Table SM 1 (below) shows the 24 different programs that were run. Each row represents a different program, and each column represents the stimulus subgroups. The letters populating the table represent where the given stimulus subgroup is present within the template Latin square.

| Program Version | Stimulus Subgroup | | | |
|---|---|---|---|---|
| | 1 | 2 | 3 | 4 |
| 1 | A | B | C | D |
| 2 | A | B | D | C |
| 3 | A | C | B | D |
| 4 | A | C | D | B |
| 5 | A | D | B | C |
| 6 | A | D | C | B |
| 7 | B | A | C | D |
| 8 | B | A | D | C |
| 9 | B | C | A | D |
| 10 | B | C | D | A |
| 11 | B | D | A | C |
| 12 | B | D | C | A |
| 13 | C | A | B | D |
| 14 | C | A | D | B |
| 15 | C | B | A | D |
| 16 | C | B | D | A |
| 17 | C | D | A | B |
| 18 | C | D | B | A |
| 19 | D | A | B | C |
| 20 | D | A | C | B |
| 21 | D | B | A | C |
| 22 | D | B | C | A |
| 23 | D | C | A | B |
| 24 | D | C | B | A |
